# Achieving universal health care coverage: Current debates in Ghana on covering those outside the formal sector

**DOI:** 10.1186/1472-698X-12-25

**Published:** 2012-10-29

**Authors:** Gilbert Abotisem Abiiro, Di McIntyre

**Affiliations:** 1 Department of Planning and Management, University for Development Studies, Box 520, Wa, Ghana; 2 Health Economics Unit, School of Public Health and Family Medicine, University of Cape Town, Anzio Road, Observatory, 7925, South Africa

**Keywords:** Universal health care coverage, National health insurance, Policy objective, Policy options, Those outside formal sector employment, Tax funding, One-time premium payment, Ghana

## Abstract

**Background:**

Globally, extending financial protection and equitable access to health services to those outside the formal sector employment is a major challenge for achieving universal coverage. While some favour contributory schemes, others have embraced tax-funded health service cover for those outside the formal sector. This paper critically examines the issue of how to cover those outside the formal sector through the lens of stakeholder views on the proposed one-time premium payment (OTPP) policy in Ghana.

**Discussion:**

Ghana in 2004 implemented a National Health Insurance Scheme, based on a contributory model where service benefits are restricted to those who contribute (with some groups exempted from contributing), as the policy direction for moving towards universal coverage. In 2008, the OTPP system was proposed as an alternative way of ensuring coverage for those outside formal sector employment. There are divergent stakeholder views with regard to the meaning of the one-time premium and how it will be financed and sustained. Our stakeholder interviews indicate that the underlying issue being debated is whether the current contributory NHIS model for those outside the formal employment sector should be maintained or whether services for this group should be tax funded. However, the advantages and disadvantages of these alternatives are not being explored in an explicit or systematic way and are obscured by the considerable confusion about the likely design of the OTPP policy. We attempt to contribute to the broader debate about how best to fund coverage for those outside the formal sector by unpacking some of these issues and pointing to the empirical evidence needed to shed even further light on appropriate funding mechanisms for universal health systems.

**Summary:**

The Ghanaian debate on OTPP is related to one of the most important challenges facing low- and middle-income countries seeking to achieve a universal health care system. It is critical that there is more extensive debate on the advantages and disadvantages of alternative funding mechanisms, supported by a solid evidence base, and with the policy objective of universal coverage providing the guiding light.

## Background

Member states of the World Health Organisation (WHO) committed themselves in 2005 to implementing universal health systems [1,2]. A universal health system aims to ensure that all residents have adequate access to needed health care without being required to make (substantial) out-of-pocket payments for health care at the time of illness [2,3]. Health care financing systems that pool resources and risk through taxes and/or mandatory insurance contributions are the main instruments for achieving universal coverage in health care [2,4].

Ghana made a bold move in introducing a National Health Insurance Scheme (NHIS) in 2004, and has made considerable progress towards the goal of universal coverage. A key challenge facing Ghana, and many other low- and middle-income countries [5], is how to expand coverage to everyone given the large proportion of the population outside of the formal employment sector. This population includes those who work in the informal sector (e.g. street vendors, minibus taxi drivers), subsistence farmers and those who are not involved in any productive economic activities. There has been heated debate on this issue within Ghana [6,7] and the current government has proposed introducing a ‘one-time NHIS premium payment (OTPP) policy’ as a means of providing financial protection to those outside the formal sector.

There are divergent approaches internationally as to how best to pursue universal coverage in countries with small formal employment sectors. On the one hand, some favour “contributory schemes” where every household is expected to contribute to an insurance scheme (as in Ghana, Rwanda, the Philippines and Vietnam), while others (such as Thailand) entirely tax fund health service cover for all those outside the formal sector [5]. There is, however, consensus that where a contributory approach is adopted, general tax funds (and/or sometimes donor funds or innovative financing such as taxes on financial transactions, airline tickets, mobile phone companies and unhealthy foods or diaspora bonds) are required to fund contributions for the poor and other vulnerable groups. The key distinction between the two approaches is that a contributory system only offers health service benefits to those who contribute (whether the contribution is made by the individual directly or on their behalf from tax or donor funds) whereas tax funding creates an entitlement to service benefits for all outside the formal employment sector.

The core of the debate is the need to generate revenue to fund health services from as broad a population base as possible. In some contexts, there is a commonly held view that those engaged in informal sector activities are able to (i.e. have sufficient income) and should (i.e. a value judgement) contribute to this revenue pool, and that the burden of funding health services should not be placed entirely on formal sector workers.

This paper critically examines the issue of how to cover those outside the formal sector through the lens of stakeholder views on the proposed OTPP in Ghana, in order to contribute to current debates on the national health insurance reforms in Ghana and broader international debates on covering those outside the formal sector. This is because stakeholder opinions are essential ingredients for understanding the political dynamics surrounding a policy issue and hence the feasibility of implementing a proposed policy. The paper draws on data collected between November 2010 and February 2011 through 28 in-depth interviews with politicians, technocrats, academics and labour unions in Accra, health workers and staff of two District Mutual Health Insurance Schemes (DMHIS), one in the north and the other in the south of the country, 6 focus group discussions with intended beneficiaries in the two districts, and a review of media reports on the policy issue (see [8] for a full description of the methods and results of the stakeholder analysis).

## Discussion

### Historical context of health care financing in Ghana

During the colonial period, health care in Ghana was funded through out-of-pocket payments [9]. This restricted access to modern health services to a privileged minority because most people could not afford the fees. Immediately after independence in 1957, health care in all public facilities was fully funded from general tax revenue [10]. This tax-funded system was not sustained due to economic recession in the 1970s, which negatively affected government revenue and spending on health care. It was therefore abandoned in favour of the introduction of nominal user fees in the early 1970s. Substantial fee payments at the point of service were introduced in 1985, popularly known as the “cash-and-carry” system, based on a loan conditionality imposed by the International Monetary Fund and the World Bank under a Structural Adjustment Programme (SAP) [11,12]. Under the “cash-and-carry system”, exemptions were introduced for the aged, pregnant women and the very poor, but they were ineffectively implemented [13,14]. Community-based health insurance schemes emerged in the 1990s to cover user fees but they had limited population coverage [15].

In 2004, arising from an election campaign promise, a mandatory National Health Insurance Scheme (NHIS) was introduced by the then ruling NPP to replace out-of-pocket payments for health care, with the ultimate goal of achieving universal coverage [16]. The NHIS is designed as a contributory system, i.e. all Ghanaians are expected to make some form of insurance contribution (although some are subsidised or exempted) and only those who contribute can benefit from the NHIS.

The NHIS contribution of most formal sector worker’s takes the form of a monthly deduction equivalent to 2.5% of the payroll from the worker’s contribution to the Social Security and National Insurance Trust (SSNIT) pension fund [10]. Though some formal sector workers such as some university employees are on a different pension scheme and hence are not members of SSNIT, the majority of formal sector workers in Ghana are SSNIT contributors. These SSNIT contributors have been assured through section 78 (3) of the NHIS Act (Act 650) that the deductions from their pension fund contributions will not affect their future pension payment [10,17]. The pension payment will be based on the full 17.5% of payroll contributions to SSNIT and not the remaining 15% after the 2.5% deduction for the NHIS. In effect, the SSNIT component could be described as a form of loan to government, although given that SSNIT has a surplus at present, it is unclear whether there will be a need for repayment or whether this can be viewed as an absolute health insurance premium by formal sector workers belonging to SSNIT. Apart from a token GHÂ¢ 4 NHI registration renewal fee paid annually by every NHIS member, SSNIT contributors are exempted from making direct premium payments to their District Mutual Health Insurance Schemes (DMHIS) with which all Ghanaians are required to register. Only non-SSNIT contributors, comprising mainly those outside the formal sector and the few formal sector workers who are not covered by the SSNIT pension fund, are required to make an annual premium contribution to their DMHIS before they are covered by the NHIS. The premiums are supposed to be structured according to ability-to-pay, ranging from GH¢ 7.20 for the lower to GH¢ 48 for the upper socio-economic groups. However, because of difficulties in assessing household income levels, many DMHIS have fixed the premium as a flat figure within this range [18]. Children below 18 years, the aged (70+), pregnant women, SSNIT pensioners and indigents are entitled to NHI membership while being exempted from premium payments, though the implementation of these exemptions appears to have been poor.

The contributions via SSNIT and premium payments by non-SSNIT formal sector workers and those outside the formal employment sector account for a relatively small share of NHIS revenue (SSNIT contributions account for 23% of revenue and premiums from non-SSNIT formal sector workers and those outside the formal sector for 5% of revenue). About 70% of the NHIS funds come from a 2.5% National Health Insurance (NHI) levy placed on all goods and services that attract valued added tax (VAT) [19,20]. Thus, in reality the NHIS is largely tax-funded. Although every Ghanaian contributes to the NHIS through the NHI levy, those households outside the formal sector who are not able to pay the annual insurance premium and who are not granted a premium exemption do not have the opportunity of benefiting from the NHI levy and other government revenue channelled to the NHIS. A number of studies have revealed that the poor and informal sector workers are less frequently enrolled in the NHIS [6,21-25], partly because of the requirement to pay an annual premium [25]. This raises questions about equity in the current operation of the NHIS and the ability of the scheme to achieve universal coverage if the annual premium is maintained.

### The proposed one-time NHIS premium payment (OTPP) policy

In 2008, the current ruling NDC party (which was the opposition party when the NHIS was introduced) made an election promise to implement a universal health insurance system which “*will guarantee access to free health care in all public health institutions”*[26]. A one-time premium payment (OTPP) system was proposed to achieve this policy objective. Since there is currently no formal policy proposal in the public domain, the NDC manifesto remains the main official document on this policy. The policy has been very controversial and highly politicised within Ghana and internationally [6-8].

### OTPP debate

Evidence from our recent stakeholder analysis [8] indicates that there is no consensus among stakeholders with regard to the meaning of the one-time premium or how the OTPP will be calculated. Some, mainly politicians of the ruling party, civil society organisations and the population outside the formal employment sector, argue that a one-time premium should require Ghanaians to pay a once-off token amount that is not significantly higher than the current annual premium level, in order to benefit freely from health care for their entire lifetime. This implies that health care will be almost fully funded from tax revenue. However, other stakeholders (mainly opposition politicians, technocrats and some academics) argue that since the manifesto stated that it would be a premium *(one-time premium),* then it means that an actuarially determined premium [27] based on the net present value (NPV) of all future premiums will have to be calculated for a single life-time payment [28]. Our study shows that few stakeholders are likely to support an OTPP based on the NPV of lifetime NHIS contributions, because it will not be affordable to most households outside the formal sector [8].

This indicates that stakeholders see the OTPP policy as representing two broad policy options, which are in line with the different international approaches to covering those outside the formal sector: 1) removing the current premiums for those outside the formal sector (and potentially non-SSNIT formal sector workers) and fully tax-funding (potentially using indirect taxes) service benefits for this group; and 2) maintaining the contributory NHIS model.

Although the detailed interviews with stakeholders allow us to distill that the underlying debate relates to contestation between these two alternative financing approaches [8], the public face of the debate focuses on the confusion created by the name given to the policy proposal, in the sense that most stakeholders are asking “what does a one-time *premium* payment mean”. The stakeholder interviews also indicate that a key factor underlying opposition to the OTPP proposal by some stakeholders is that it could represent a move away from what is seen as the already entrenched policy direction of a *contributory* scheme. For example, one academic who was interviewed stated: *“I don’t understand it … it is a political nonsense. It doesn’t conform to any health insurance. If it is a tax-based system, I would understand it but not under the National Health Insurance System”*. Similar views were expressed by other interviewees, such as an opposition politician who indicated that: “*from my personal understanding, one-time premium payment is really not insurance; if it is just about paying a registration fee then that becomes like a National Health Service, akin to the British”*.

There are examples of countries which have changed course in their health care financing policies. For example, Thailand changed from a contributory system for covering those outside the formal employment sector to tax funding services for this group [5]. In order to assess whether such a shift is appropriate or not in the Ghanaian context, it may be helpful to consider explicitly the advantages and disadvantages of these alternative funding approaches from the perspective of key stakeholders, and to identify what evidence is needed to provide an empirical basis for policy decision-making.

### Key issues in critically evaluating alternative approaches for covering those outside the formal sector in Ghana

From the evidence gathered in our recent stakeholder analysis [8] and a review of existing literature, the following potential advantages and disadvantages can be raised about the alternative approaches to funding services for those outside the formal employment sector within the Ghanaian context. While there are a small number of formal sector workers who are not members of SSNIT (such as some university employees) and are therefore also required to pay premiums directly to the DMHIS, we are focusing here on those outside the formal employment sector.

If the current contributory premium system is maintained, the following may be the advantages:

•It will continue to provide an additional source of revenue for the NHIS.

•The collection of the annual premium is a source of employment for some people (premium collectors).

•Paying a premium may instil in clients a sense of ownership of the scheme and make them individually feel responsible for their health and health care.

However, the disadvantages of maintaining the current system include:

•The collection of premiums from the informal sector in Ghana is time-consuming, expensive and sometimes associated with fraud on the part of premium collectors [25], yet informal sector premiums only constitute about 5% of NHIS revenue [20].

•The requirement of paying a premium for enrolment denies those outside the formal sector who cannot afford these payments and cannot secure premium exemptions access to health care and benefits from the NHI levy and other tax subsidies to the NHIS.

•These contributions are very regressive since they are often fixed at a flat amount and hence impose a higher burden of NHIS payments on the poor [12]. For instance, the bottom 20% of the population contributes 3.85% of their consumption expenditure as informal sector premiums while the top 20% contributes only 0.27% [25].

On the other hand, the arguments that can be advanced for tax-funded cover for those outside the formal sector include:

•It will promote equity in financing as tax funding is progressive in Ghana (not only personal income taxes but also most indirect taxes, including VAT) [18,25]. For instance, the poorest 20% of the Ghanaian population spends only 2% of their consumption expenditure on VAT, while the richest 20% spends 3.5% of their consumption expenditure on VAT [25]. It will also promote equity in access to health care as all will have financial protection against the burden of the “cash and carry system” which still faces those who are not members of the NHIS.

•Taxes are easier to collect than insurance premiums as tax collection mechanisms are already well established. For example, there are already mechanisms for collecting VAT and other indirect taxes in Ghana while income taxes are directly deducted from the payroll.

Its disadvantages however include:

•Ghanaians are often not willing to accept additional (general) taxation [29]. In contrast, people may be more likely to accept payment of insurance premiums from which they receive specific health service benefits [30,31].

•It may be difficult to sustain a largely tax-funded system in Ghana in the context of high population growth and associated increasing demand on publicly funded health services, economic instability and citizens’ lack of trust in the continued existence of political commitment to tax funding of health services.

•An inevitable increase in utilisation of health care under a largely tax-funded system in the midst of the current limited health care facilities and personnel will increase the workload of providers. This can lead to overcrowding at health facilities, delays in seeing patients, poor attitude of health providers towards clients and hence, can negatively affect the quality and efficiency of health service provision in Ghana.

Empirical evidence, such as quantifying the costs of collecting insurance premiums from those outside the formal sector to assess the net revenue generation from the current contributory system, and the revenue generating potential of different forms of tax, would contribute to a constructive debate on how best to fund a universal health system in Ghana. It is important to recognise that some of the issues raised above, such as utilisation increases and inadequate staffing levels, will exist irrespective of whether health care is funded from insurance premiums or taxes. Such issues therefore need to be addressed through other policy instruments; for example, addressing utilisation increases through effective primary care gate-keeping.

In engaging in this debate, it is important to acknowledge that the core objective of the current insurance contributions is to raise revenue from the informal sector. Given that there are other ways of generating revenue from this group, for example through indirect taxes, what would be the best way to raise this revenue? From an efficiency point of view, it is undoubtedly more efficient (in terms of net revenue generation after taking account of collection costs) to collect indirect taxes from the informal sector than insurance premiums. Household survey data can be used to identify the goods and services purchased by those in the informal sector who could be regarded as being able to contribute to funding health care but are not widely used by poor households so as to better target an indirect tax that could be dedicated to health care funding. From an equity perspective, is it really feasible and administratively efficient to identify those who are unable to afford insurance premiums in order to exempt them and provide financial protection and needed care to all residents or it is more feasible to collect revenue through other mechanisms (e.g. indirect taxes) from those in the informal sector with the ability-to-pay? It also needs to be explored whether collecting informal sector contributions through (earmarked) indirect taxes will really have an impact on (ownership) perceptions of the health system if residents are aware that the taxes paid by them are used to cater for their health care.

Even if these issues were thoroughly explored and considered, a key remaining issue would be the financial feasibility and sustainability of moving towards tax funding of cover for those outside the formal sector. The lack of an explicit indication of how the OTPP policy would be funded, and lack of evidence that this funding mechanism could generate the required resources, is possibly the greatest deficiency in the OTPP policy proposal. Ghana has led the way in demonstrating the revenue generating power of dedicated indirect taxes, through its 2.5% VAT levy which generates 70% of the revenue of the NHIS. However, it is not clear whether a further increase in VAT will be possible, whether oil revenues could be used to co-fund the NHI or whether other indirect taxes or innovative financing options (e.g. such as levies on large and profitable companies as with the 10% levy on mobile phone companies in Gabon [2]) could be explored.

Finally, it is important to recognise that improving equity and efficiency in financing will not by itself translate into universal access to needed health care; additional efforts are required to reduce physical barriers to access (e.g. through increasing the number of primary care facilities, increasing staffing levels and improving the routine availability of essential medicines within facilities). Thus, the current debate about the OTPP is only one aspect of the challenges facing Ghana in its highly regarded efforts to pursue universal coverage.

## Summary

The Ghanaian debate on OTPP is clouded by the confusion about the potential content of the policy. Stakeholder interviews indicate that the underlying issue in the debate about this policy is whether the current contributory NHIS model for those outside the formal sector should be maintained or the annual premiums should be removed so that health care is largely funded from (potentially earmarked indirect) taxes. Switching to an earmarked tax-funded system may have greater prospects for achieving universal coverage in Ghana, but, as pointed out by some, the current Ghanaian economic environment may not be favourable for its immediate implementation [7]. Nevertheless, there would be value in assessing whether it should at least be considered as the strategic direction of the NHIS. Other funding sources such as oil revenues and/or innovative financing mechanisms could also be considered.

The OTPP debate also touches on issues of social values in Ghana. It points to an underlying debate about whether poor people should have some personal direct financial responsibility for their health care (arguments for an annual premium or an actuarially determined single premium) versus the right to health care for poor people without the direct payment of a premium (arguments for indirect tax-based financing that subsidizes healthcare for all regardless of income status).

The challenge of how best to ensure that those outside the formal sector are provided with financial protection and access to needed care is not unique to Ghana. It is possibly the most important issue facing low- and middle-income countries wishing to pursue universal coverage. It is critical that there is more extensive debate on the advantages and disadvantages of alternative approaches to funding services for this group, supported by a solid evidence base, and with the policy objective of universal coverage providing the guiding light.

## Abbreviations

DMHISs: District Mutual Health Insurance Schemes; GH¢: Ghana cedi; IMF: International Monetary Fund; MOH: Ministry of Health; NDC: National Democratic Congress; NHI: National Health Insurance; NHIA: National Health Insurance Authority; NHIS: National Health Insurance Scheme; NPV: Net Present Value; OTPP: One-time Premium Payment policy; SAP: Structural Adjustment Programme; SSNIT: Social Security and National Insurance Trust; VAT: Value Added Tax; WHO: World Health Organisation.

## Competing interest

We declare that we have no competing interest.

## Authors' contributions

GA conceptualised and designed the study, undertook the data collection and analysis, and drafted the paper. DM supported the conceptualisation and design of the study, data analysis and paper drafting, and critically reviewed the drafts and contributed to its finalisation. Both authors read and approved the final manuscript.

## Pre-publication history

The pre-publication history for this paper can be accessed here:

http://www.biomedcentral.com/1472-698X/12/25/prepub
